# Decision tree-based learning and laboratory data mining: an efficient approach to amebiasis testing

**DOI:** 10.1186/s13071-024-06618-6

**Published:** 2025-01-29

**Authors:** Enas Al-khlifeh, Ahmad S. Tarawneh, Khalid Almohammadi, Malek Alrashidi, Ramadan Hassanat, Ahmad B. Hassanat

**Affiliations:** 1 Department of Applied Biology, Al-Balqa Applied University, Salt, Jordan; 2 Faculty of Information Technology, Mutah University, Mutah, Jordan; 3 Computer Science Department, Applied College, University of Tabuk, Tabuk, Saudi Arabia; 4 General Surgery Department, Jordanian Royal medical service, Amman, Jordan

**Keywords:** Amebiasis, *E. histolytica*, Electronic medical records (EMR), Microscopic diagnosis, Machine learning, Feature selection, Decision tree, Stool RBCs, Leukocytosis, Jordan

## Abstract

**Background:**

Amebiasis represents a significant global health concern. This is especially evident in developing countries, where infections are more common. The primary diagnostic method in laboratories involves the microscopy of stool samples. However, this approach can sometimes result in the misinterpretation of amebiasis as other gastroenteritis (GE) conditions. The goal of the work is to produce a machine learning (ML) model that uses laboratory findings and demographic information to automatically predict amebiasis.

**Method:**

Data extracted from Jordanian electronic medical records (EMR) between 2020 and 2022 comprised 763 amebic cases and 314 nonamebic cases. Patient demographics, clinical signs, microscopic diagnoses, and leukocyte counts were used to train eight decision tree algorithms and compare their accuracy of predictions. Feature ranking and correlation methods were implemented to enhance the accuracy of classifying amebiasis from other conditions.

**Results:**

The primary dependent variables distinguishing amebiasis include the percentage of neutrophils, mucus presence, and the counts of red blood cells (RBCs) and white blood cells (WBCs) in stool samples. Prediction accuracy and precision ranged from 92% to 94.6% when employing decision tree classifiers including decision tree (DT), random forest (RF), XGBoost, AdaBoost, and gradient boosting (GB). However, the optimized RF model demonstrated an area under the curve (AUC) of 98% for detecting amebiasis from laboratory data, utilizing only 300 estimators with a max depth of 20. This study highlights that amebiasis is a significant health concern in Jordan, responsible for 17.22% of all gastroenteritis episodes in this study. Male sex and age were associated with higher incidence of amebiasis (*P* = 0.014), with over 25% of cases occurring in infants and toddlers.

**Conclusions:**

The application of ML to EMR can accurately predict amebiasis. This finding significantly contributes to the emerging use of ML as a decision support system in parasitic disease diagnosis.

**Graphical Abstract:**

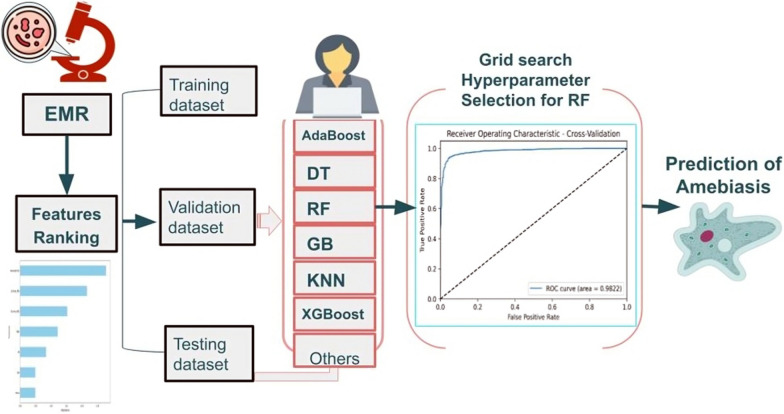

**Supplementary Information:**

The online version contains supplementary material available at 10.1186/s13071-024-06618-6.

## Background

More than a million people are impacted by food and waterborne parasites each year, despite massive international efforts to control and prevent their spread [1]. *Entamoeba histolytica* is a protozoan parasite that infects humans and causes two forms of pathology: intestinal amebiasis (or amoebic dysentery) and amoebic liver abscesses, parasitic diseases most frequently associated with fatalities globally [2]. Determining the epidemiology of amebiasis is challenging because of its silent nature and technical difficulties. There may be overlap between symptoms of amebiasis and other GE diseases, resulting in variable estimates of the prevalence [3], ranging from approximately 5% [4] to as high as 40% [5].

Amebiasis remains endemic in several developing countries, particularly those with low economic level and high population density. For instance, pathogenic *E. histolytica* has been found in 22 of 30 American countries, with an overall prevalence of 9% [6]. Amebic infections are particularly prevalent in regions with tropical or subtropical climates, such as Latin America, where 22% of Brazilians have *Entamoeba* spp. [3], and 28–42% of patients in rural Mexico were found to have pathogenic *E. histolytica* in a seroprevalence investigation [5]. However, despite being subtropical, only a small percentage of the North Indian population (5%) is affected by *E. histolytica* [4]. In Asia, a 10-year retrospective study conducted in Taiwan discovered that 16.1 out of every 1,000,000 people develop amebiasis [7].

Middle Eastern countries are considered key endemic foci of amebiasis owing to their large young population and inadequate sanitation and water systems in many areas [8]; however, variations are found in patient records. For instance, amebiasis is low in Iran [9], whereas in Erbil City, northern Iraq, pathogenic *E. histolytica* accounted for 81.4 % of asymptomatic *Entamoeba* infections, with an overall frequency of 6% [10]. Furthermore, in Saudi Arabia up to 83% of adult patients in Jeddah City [11] and 40.7% in Najran City [12] had intestinal infections caused by *E. histolytica*. In patients from different parts of Jordan, Giardia lamblia and *E. histolytica*/*E. dispar* were shown to be the most common intestinal parasites. The southern region of Jordan had the highest prevalence (80.7%) [13], while hospitals in the Jordanian city of Amman and its northern region reported prevalence rates of 27.81% and 33%, respectively [14, 15]. Amebiasis has also been detected in specific Jordanian populations. For example, patients with GE symptoms have an *E. histolytica* infection rate of 10% in Bedouin tribes [16]. In contrast, only one such case has been documented among culinary hotel employees [17]. Overall, amebiasis is endemic, according to previous studies carried out in Jordan. But there are still several unresolved questions. The degree of amebiasis in Jordanian patients of all ages and sexes is unclear, in addition to the clinical and laboratory symptoms.

Frequent episodes of diarrheal to semisoft stools, often containing blood, mucus, and live trophozoites, are hallmarks of amebic dysentery [18]. Abdominal pain can range from slight soreness to intense discomfort. Numerous investigations have documented adverse systemic effects, including elevated leukocyte counts [19, 20]. Amoebic colitis is often accompanied by abdominal soreness, and in some cases, peritonitis or toxic megacolon can exacerbate fulminant colitis [21]. Persistent amebic infection in the colon can also mimic inflammatory bowel disease [22]. Additionally, amoebic dysentery might be mistaken for other causes of GE conditions, including ulcerative colitis, salmonellosis, or shigellosis [23]. Compared with bacillary dysentery, amoebic dysentery typically results in less frequent and less watery stools. Amebiasis is characterized by the presence of mucus and blood specks [24]. However, symptoms of shigellosis often include bloody diarrhea. It is also noted that complete blood count (CBC) measures overlap between amebiasis and other GE cases [25]. In fact, it remains difficult to distinguish amebiasis from other causes of GE as a result of these variables.

Testing for amebiasis involves a variety of laboratory techniques, beginning with simpler methods such as microscopy, and advancing to more sophisticated methods such as molecular diagnostics for detecting parasite DNA in feces, immunoassays involving serology, and direct antigen identification [26]. Sometimes, combining multiple tests can yield conclusive results. Although rapid antigen testing is available, it may not effectively distinguish between an acute infection and a past infection [26], especially in populations where the frequency of infection is high [27]. In Jordan, as in many other countries, the primary method used to diagnose amoebic and other infectious gastroenteritis is the traditional microscopic examination. However, microscopic inspection requires qualified personnel. Sensitive and specific immunoassays can detect *E. histolytica* antigens in feces, thus confirming the diagnosis. However, the majority of public health sector laboratories in Jordan and many other countries lack the immunoassay equipment necessary to confirm infection. This method is limited to Private sector laboratories, which are not fully accessible to all patients owing to insufficient health insurance coverage. In such cases, integrating electronic system techniques with medical data may offer solutions to diagnostic challenges.

Artificial intelligence (AI) and machine learning (ML) are increasingly used in the medical industry. Serving as a diagnostic decision support system, for instance [28]. Machine learning has plenty of possibilities for diagnosing infectious diseases. Machine learning and deep learning (DL) models have been applied to parasitology to predict disease, detect parasites [29], and identify risk factors [30]. However, these applications are limited, with most techniques related to malaria [31], trypanosomiasis [32], and toxoplasmosis [33]. Besides, few published studies have discussed the use of ML for other protozoal infections [34], and none have included the prediction of amebiasis. However, since it is impossible to pinpoint the exact cause of GE based solely on clinical features, AI techniques based on laboratory data processing for infectious GE are a crucial field of study. An amoebic trophozoite with characteristic morphology and red blood cell ingestion must be found in a fresh stool specimen to confirm an official diagnosis of invasive amebiasis. Even skilled technicians, meanwhile, commonly confuse partially digested food particles, WBC, hematophagous macrophages, and nonpathogenic protozoa for amoebic trophozoites. The application of AI has the potential to result in accurate evaluations being performed automatically, which in turn reduces errors and times in diagnosis and improves performance in prediction and detection. This is especially valid when considering amebiasis.

The main goal of this study is to model the identification of amebiasis based on laboratory results and subsequent separation from other GE causes using machine learning. In particular, our objectives are:

1. Assess the current state of amebiasis in Jordan’s Al-Salt Province.
2. Examine the key features that set amebiasis apart from other GE instances. These features are directly generated from clinical, demographic, hematological, and microscopic data.
3. Examine how well decision tree classifiers work with EMR data to identify amebiasis.
4. Verify the outcomes of the selected model (the best performer) by repeatedly using a holdout dataset with different sizes. This makes it more likely that the model will function as anticipated in the actual world and generalize effectively to new unseen inputs.

This study presents, for the first time, the use of feature selection and ML-based decision tree classifier approaches in the context of amebiasis detection from structured data.

## Methods

### Study environment

The targeted population inhabits Al-Salt, one of Jordan’s largest cities, which is located north of Amman. In this study, we looked at parasitology data from patients at Al-Hussein Hospital, Al-Salt, the city’s only hospital, which is also a government-run university hospital. Thus, the statistics may be viewed as indicative of the 180,090-person majority population of the city, which consists of 51% males and 49% females.

### Description of the dataset

Between 7 September 2020 and December 2022, information was compiled from Jordan’s Hakeem electronic health record system [35]. The data used for the analysis included wet-mount reports of the detection of intestinal parasites. Laboratory tests were performed by the normal operating procedures of the Jordanian health centers. Briefly, stool samples were collected in labeled, clean, dry, leak-proof, and sterile plastic containers for a laboratory diagnosis from all patients with GE with suspected involvement of an intestinal parasite. Within 30 min of sample collection, direct stool examinations were performed to examine for the presence of common intestinal parasites (*E. histolytica*/*dispar*, *Giardia lamblia*) and worms (*Ascaris lumbricoides*, *Hymenolepis nana*, *Enterobius vermicularis*, *Strongyloides stercoralis*, and *Trichuris trichiura*, hookworms besides *Taenia*, and other cestode species). After stool examinations, the infected patients were treated according to the national guidelines.

Positive parasitology results for *Entamoeba* species (*n* = 763) were included in this study, as it is the predominant protozoan reported. A few sporadic cases of *Giardia* (fewer than 20) were reported during the study period; however, they were not included in our analysis. For ML engineering, we added 314 more examples to our analysis. Those instances exhibited GE symptoms, but parasites were not microscopically detected in them.

Hakeem’s sociodemographic data and laboratory results were examined and compiled using a worksheet designed for this purpose. The dataset contained the date, sex, age (years), diagnosis (diarrheal, semisoft stool, and abdominal pain without diarrhea), and stool examination results with severity indicators such as stool–(RBCs), and leukocytosis or white blood cell (WBC) counts. The WBC count was categorized into three groups based on the number of cells/high-power field: low = 0–3; medium = 3–6; and high = more than 6. Additionally, complete blood count (CBC) results were used to obtain WBC and neutrophil counts because they are considered signs of a reaction to the infection and its severity. The patients were categorized into four age groups: toddlers (less than 5 years), school-going age (represents 5–18 years), active workforce (19–59 years old), and adults (older than 60 years).

**Table 1:** Summary statistics for amebiasis and non-amebiasis

Amebiasis								
Feature	Count	Mean	Std	Min	25%	50%	75%	Max
Age (years)	763	27.90433	25.22055	1	4	22	48	99
Hb (g/dL)	763	12.78657	1.878827	7.2	11.8	12.79	13.9	17.7
WBC	763	9.220616	3.579502	1.06	7.07	8.51	10.42	31.2
Neutrophils (%)	763	0.659961	0.155015	0.11	0.61	0.64	0.76	0.98

Non-amebiasis								
Feature	Count	Mean	Std	Min	25%	50%	75%	Max
Age (years)	314	24.58917	24.91391	1	1	16.5	44	92
Hb (g/dL)	314	11.90831	1.858182	6.5	10.9	12.16	13.2	16.5
WBC	314	10.93067	3.096141	3.2	8.7	11.2	13.275	22.4
Neutrophils (%)	314	0.400247	0.214546	0.04	0.2025	0.38	0.57	0.885

Tables 1 and  2 provide a statistical summary of observations in each class of the dataset. Table 1 presents the summary statistics for the numerical features of the amebiasis and non-amebiasis cases. Similarly, Table 2 shows the frequency distribution of categorical features by their respective values.

As one can see from Table 1 presents the summary statistics for key features in amebiasis and non-amebiasis cases. For the amebiasis class, the average age is 27.9 ± 25.22 years, with hemoglobin (Hb) levels averaging 12.79 ± 1.88 g/dL and WBC at 9.22 ± 3.58. The neutrophil percentage in this class averages 65.99 ± 15.5%. In contrast, the non-amebiasis class has a slightly younger average age of 24.59 ± 24.91 years, with a lower mean Hb level of 11.91 ± 1.86 g/dL and a higher mean WBC count of 10.93 ± 3.10. The neutrophil percentage in this class is lower, averaging 40.02 ± 21.45%. These differences in hematological parameters may suggest variations in immune response between the two classes.

**Table 2:** Summary of categorical variables by count and percentage

Feature	Values	Amebiasis		Non-amebiasis	
		Counts	Percentage	Counts	Percentage
Sex	F	382	50.07	169	53.82
	M	381	49.93	145	46.18
Diagnosis	Diarrhea	426	55.83	193	61.46
	Semisoft	189	24.77	121	38.54
	Abdominal pain	145	19.00	0	0
	Rectal bleeding	3	0.39	0	0
Diarrhea-RBC	Low	123	16.12	101	65.61
	Moderate	188	24.64	237	32.17
	High	407	53.34	4	1.27
	Very high	45	5.90	3	0.96
Diarrhea-WBC	Low	100	13.11	62	19.75
	Moderate	237	31.06	237	75.48
	High	335	43.91	6	1.91
	Very high	91	11.93	9	2.87
Mucus	Absent	558	73.13	313	99.68
	Present	202	26.47	1	0.32
	Numerous	3	0.39	0	0

Table 2 summarizes the distribution of categorical variables in both the amebiasis and non-amebiasis classes by count and percentage. In terms of sex distribution, females represent 50.1% of the amebiasis class and 53.8% of the non-amebiasis class. For diagnosis, diarrhea is the most common condition, affecting 55.8% of individuals with amebiasis and 61.5% of those without. In the diarrhea RBC category, high RBC presence is seen in 53.3% of the amebiasis class but only 1.3% in the non-amebiasis class. Regarding diarrhea-WBC counts, high levels are observed in 43.9% of amebiasis cases, while moderate levels are dominant in the non-amebiasis class (75.5%). For mucus, its absence is most frequent, particularly in the non-amebiasis class (99.7%). These variations highlight differences in symptom distribution between the two classes.

### Data processing, analysis, and visualization

After being extracted from Hakeem, the data were cleaned using Pandas Python software [36]. Numpy [37] and Scipy [38] packages were used to categorize the data, establish the association between variables, and display their distribution patterns. To compare qualitative data, a chi-squared test was performed, which clarified the distributions of categorical variables, specifically the relationship between risk factors for amebiasis, including sex, age, and other laboratory findings that indicated the severity of infection, including diarrhea or the presence of RBCs or WBCs in the stool. Correlations among age, WBC count, neutrophil count, and neutrophil percentage were tested using one-way analysis of variance. Student’s *t*-tests were used to compare numerical variables (WBC count, neutrophil count, and neutrophil percentage) and to determine significant differences between groups. The analysis followed a significance threshold of *P* < 0.05, ensuring the reliability and validity of the results. Graphical data representations were generated using Matplotlib Pyplot package [39] and Seaborn in Python [40].

For encoding categorical variables, we employed a straightforward factorization approach, assigning values such as “male” = 0, “female” = 1, “mucus present” = 1, “mucus absent” = 0, and “numerous” = 2, and so on for all categorical variables including “Diagnosis,” “Diarrhea-RBC,” and “Diarrhea-WBC.” Although we initially tested one-hot encoding in our pilot experiments, the results were not as favorable as those obtained with the factorization method. Consequently, we chose to use factorization encoding for all of our reported experiments.

### Machine learning model

The goal of this research was to create a model that can correctly identify between amebiasis and non-amebic gastrointestinal data. Classification is the process of predicting an unlabeled object’s class using its properties [28, 41]. The process consists of the following steps:

1. Training: A training dataset is utilized to develop a classification model. We used supervised learning techniques in our research as our data was labeled with both text and numerical values, and the dataset was large and varied. Parametric approaches such as logistic regression (LR) and Naive Bayes (NB) and nonparametric methods like multilayer perception neural networks (MLP), *K*-nearest neighbor (*K*NN), Hassanat *K*NN (H*K*NN), and decision trees (DT) are examples of machine learning (ML) techniques used in this study. We also looked at techniques such as support vector machine (SVM) and linear SVM (LSVM), which are classified as both parametric and nonparametric. Random forest (RF), AdaBoost, eXtreme gradient boosting (XGBoost), and gradient boosting (GB) were also used as ensemble approaches. We also used additional well-known models that fit linear regressors and classifiers under convex loss functions, such as linear discriminant analysis (LDA) and stochastic gradient descent (SGD) [42–44].
2. Testing: Using a different test dataset, this stage assesses the trained model’s effectiveness and quality. To evaluate the classifiers’ prediction accuracy and generalization capacity, the test data are fed into the same classifiers’ models that were obtained by the training phase.

We used fivefold cross-validation to test and train all of the data to assess the aforementioned ML techniques and determine which classifier best suited our data for the best identification of amebiasis  [45, 46]. Each classifier was evaluated for performance using the most common metrics (accuracy, precision, recall, and F1 score) to determine the most effective model for our data. We used the default parameters for each classifier, as the aim is to find the best classifier that suits our data rather than optimizing the performance of each, such a process should be our next step if the data are too complex to be classified by all classifiers examined.

## Results

### Feature engineering

In this study, we employed two feature selection methods: feature correlation [47] and feature importance ranking [48].

The random forest (RF) technique was employed to rank the features in the dataset under investigation on the basis of their importance concerning amebiasis. Several decision trees were built using the robust ensemble learning methodology known as the RF method, and their outputs were combined to increase prediction accuracy and reduce overfitting. In RF, the contribution of each feature to the overall model performance is used to define the feature’s relevance. To be more precise, we used the Gini index as a standard for determining the significance of each attribute. A dataset’s impurity is measured by the Gini index, which indicates the probability that an element selected at random would be incorrectly classified if its labels were distributed in accordance with the subset’s label distribution. A purer node—one in which its feature successfully divides the classes—is indicated by a lower Gini value.

A variety of factors, including clinical signs and laboratory data from the patients under study, were incorporated into our analysis. These included total and differential leukocyte counts, red blood cell and fecal leukocyte counts, and other factors including patient age and sex. Our goal was to improve the diagnosis accuracy by taking these aspects into account  [49, 50].

**Fig. 1:**
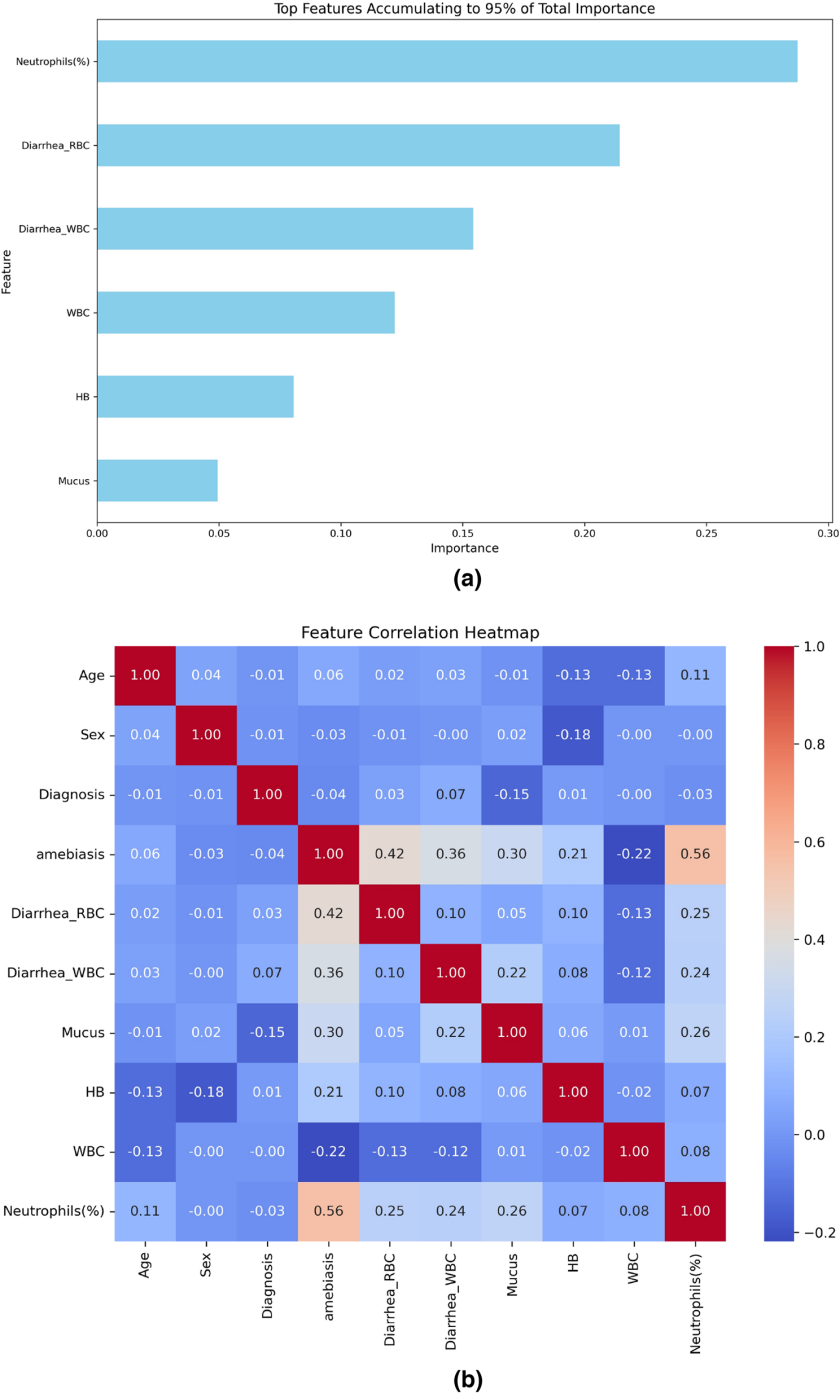
**a** The top features account for 95% of the data’s importance in predicting amebiasis. **b** A correlation heat map where strong positive correlations are denoted by red, strong negative correlations by blue, and a lack of color indicates weak correlation

Our goal was to pinpoint the most important risk factors that, when taken as a whole, contribute to 95% of the overall feature significance rating that the RF model produced. The proportion of neutrophil percentage, diarrhea-RBC, diarrhea-WBC, WBC, Hb, and mucus presence were the main parameters that were found. Figure 1a lists these parameters in decreasing order of significance.

Using this method not only helped us determine the most important amebiasis predictors but also shed light on the underlying laboratory and clinical traits linked to the illness. We can more effectively guide focused actions and clinical decision-making by concentrating on traits with high significance ratings.

The cor() function from the basic R language package is utilized to compute the correlation; by default, it computes the Pearson correlation coefficient. The Pearson correlation coefficient between variables *X* and *Y* can be calculated using the following formula:

$$r_{XY} = \frac{\sum ((X_i - \bar{X})(Y_i - \bar{Y}))}{\sqrt{\sum (X_i - \bar{X})^2 \sum (Y_i - \bar{Y})^2}}$$,

where $X_i$ and $Y_i$ are individual data points for variables *X* and *Y*, $\bar{X}$ and $\bar{Y}$ are the means of the variables *X* and *Y*, respectively.

The heatmap displayed in Fig. 1 (row 4) illustrates the Pearson correlation coefficients between all variables and the dependent variable (amebiasis), which show a significant relationship between the aforementioned top six strongest features and the microscopic diagnosis of amebiasis. While neutrophil (%), diarrhea-RBC, diarrhea-WBC, mucus, and Hb have comparatively greater positive associations (0.56, 0.36, 0.30, 0.30, and 0.21, respectively), WBC exhibits a relatively substantial negative connection (−0.22). The classification of correlation strength ($r_{XY}$) is not widely agreed upon; nonetheless, it is generally understood to be weak when $r_{XY}$ is in the range (−0.4, 0.4), moderate when $r_{XY}$ falls between 0.4 and 0.7, or −0.4 and −0.7, and strong when $-0.7> r_{XY} > 0.7$ [51].

In the context of this study, these correlations are seen as having a relative strength even if they do not meet traditional criteria for being called “strong.” Their inclusion as essential features is justified by the relative intensity of the connection with amebiasis. This connection, which is consistent with the RF results as shown by the correlation heatmap (Fig. 1), demonstrates the selection of these six variables, which collectively account for 95% of the RF classification importance.

The Pearson correlation coefficients between all variables and the dependent variable (amebiasis), which are illustrated by the heatmap shown in Fig. 1 (row 4), demonstrate that the aforementioned top six strongest features have a significant link with the microscopic diagnosis of amebiasis. WBC shows a relatively significant negative correlation (−0.22), while neutrophil (%), diarrhea-RBC, diarrhea-WBC, mucus, and Hb show relatively stronger positive relationships (0.56, 0.36, 0.30, 0.30, and 0.21, respectively). The selection of these six features, which together account for 95% of the RF classification importance, is demonstrated by this correlation, which aligns with the RF results, as seen by the correlation heatmap (Fig. 1).

**Fig. 2:**
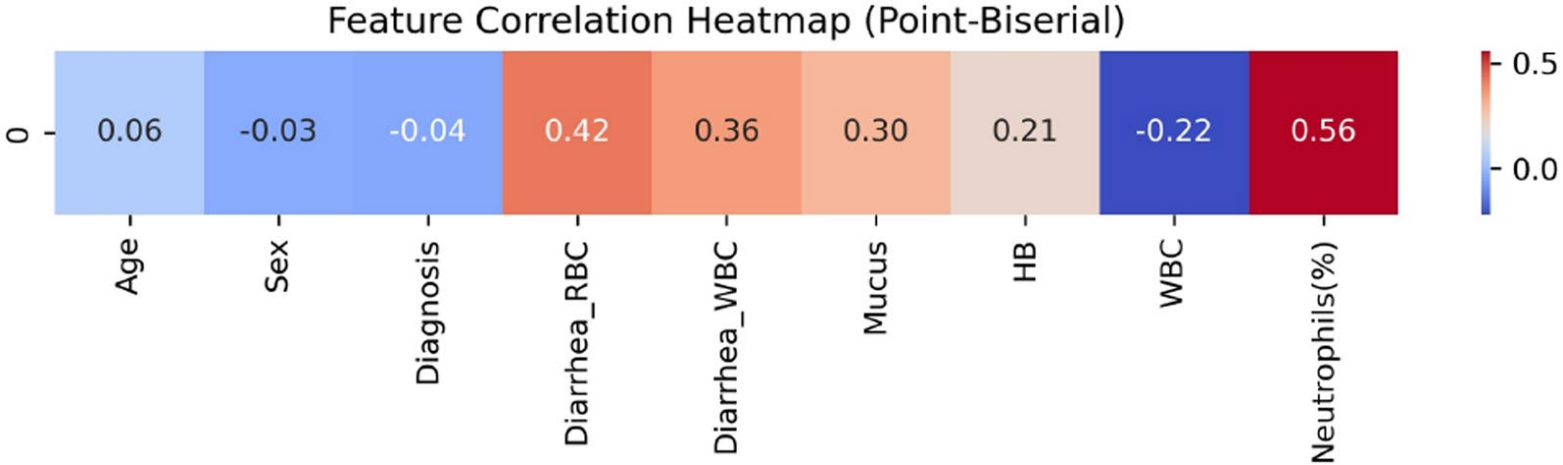
A point-biserial correlation heatmap, where strong positive correlations are denoted by red, strong negative correlations by blue, and a lack of color indicates weak correlation

Because Pearson correlation presumes that both variables are continuous and normally distributed, it is typically not advised to utilize it with binary dependent (amebiasis) and continuous independent variables (the other features). In practice, it is still occasionally utilized to quickly understand the linear connection between a continuous and binary variable. Other approaches are advised to obtain a more suitable measure of the relationship between a continuous and binary variable, such as the point-biserial correlation, a special case of the Pearson correlation in which one variable is binary [52]. However, we obtained the same coefficients using the point-biserial correlation as shown in Fig. 2.

### Amebiasis prevalence and related risk factors

**Fig. 3:**
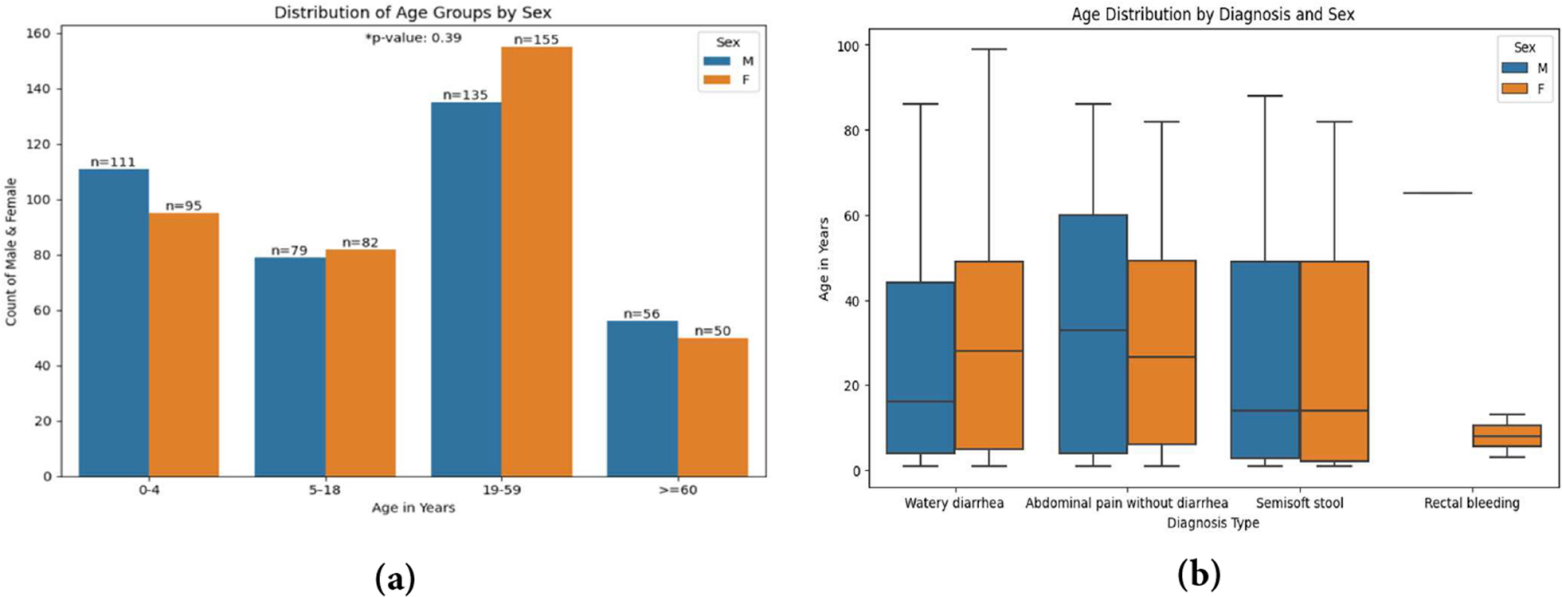
Comparison of clinical manifestations, age, and sex of the Jordanian patients with confirmed cases of amebiasis. **a** Age distribution of the cases and **b** age distribution according to symptoms being diagnosed. Significant difference when *P*-value < 0.05. *n* = 763

A total of 4429 stool tests were performed over the study period on patients with GE symptoms. Among these, 763 were confirmed to have amebiasis (17.22%), with 381 (49.9%) male and 382 (50.1%) female. The study patients’ characteristics are shown in Fig. 3. The study participants were between the ages of less than 1 month and 99 years. Male patients had a median age of 19 years, while female patients had a median age of 24 years. The median ages of males and females, however, did not differ significantly (*P* = 0.43). The prevalence of amebiasis among males and females across various age groups was not significantly different (*P* = 0.39). However, among toddlers and preschoolers, more males were observed than females (Fig. 3).

**Fig. 4:**
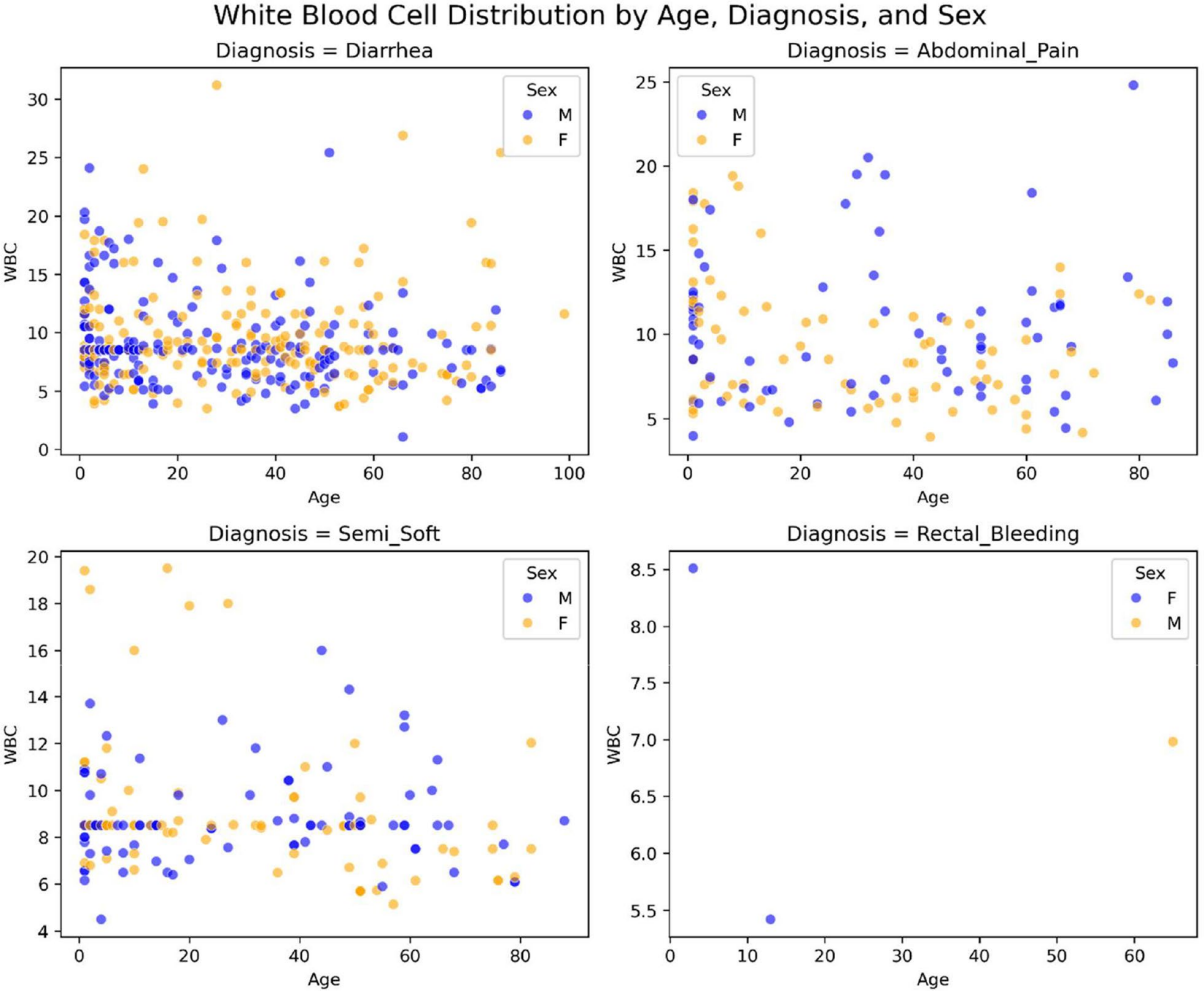
Distribution of laboratory findings, clinical manifestations, age, and sex of Jordanian patients with confirmed cases of amebiasis gastroenteritis. Severity features indicated by WBC count (*n* = 763)

**Fig. 5:**
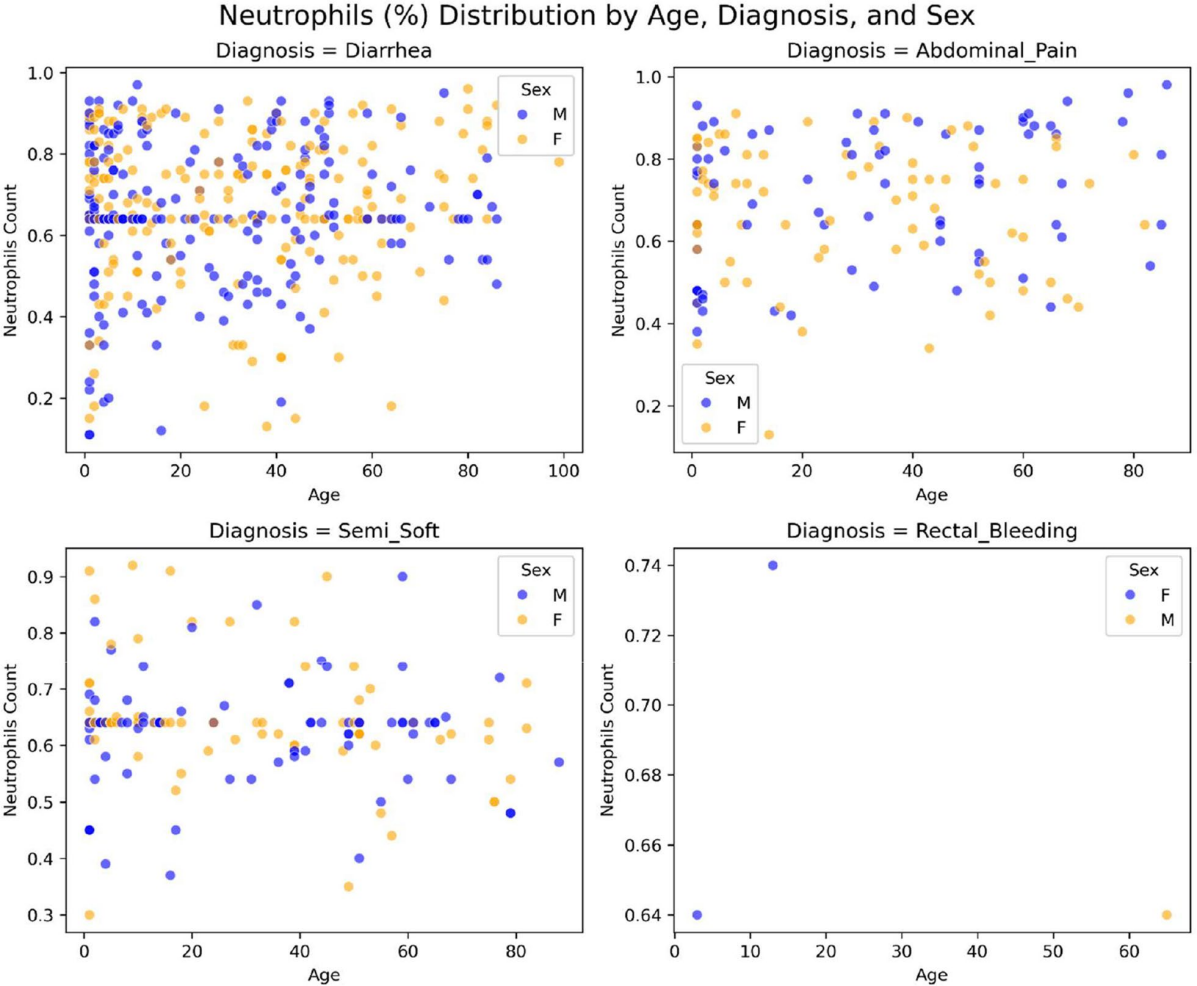
Distribution of laboratory findings, clinical manifestations, age, and sex of Jordanian patients with confirmed cases of amebiasis. Severity features indicated by neutrophil percentage (*n* = 763)

In contrast, considerably more females were observed than males in the adult group (aged 19–59 years). Male sex and age both indicated a higher incidence of amebiasis when comparing males of different ages to females of similar ages (*P* = 0.014). Among all age groups studied, adults aged 20–59 years had the highest infection rate, whereas those older than 60 years had the lowest infection rate (Fig. 4). The percentages of amoebic infections across age groups did not differ significantly (*P* = 0.06). According to the clinical signs, the intensity of the symptoms was as follows: abdominal pain without diarrhea, 145 (19%); semisoft stools, 189 (24.7%); watery diarrhea, 426 (55.8%); and rectal bleeding, 3 (0.3%) (Fig. 5).

Individuals aged 1 month to 4 years accounted for 27% of the total, with 107/206 cases (51.9%) diagnosed with watery diarrhea. As expected, WBCs and neutrophil (%) showed a correlation with the diagnosis. These measurements increased when patients were diagnosed with watery diarrhea (Fig. 5).

### Machine learning results

**Table 3:** Amebiasis prediction results ± their standard deviation along the five runs of the fivefold cross-validation

	Accuracy	Precision	Recall	F1 score
LR	0.8911 (± 0.0159)	0.8760 (± 0.0189)	0.8555 (± 0.0198)	0.8642 (± 0.0179)
SGD	0.7626 (± 0.1132)	0.7851 (± 0.0492)	0.7691 (± 0.0710)	0.7298 (± 0.0992)
SVM	0.7067 (± 0.0283)	0.3537 (± 0.0148)	0.4994 (± 0.0012)	0.4139 (± 0.0097)
LSVM	0.8641 (± 0.0452)	0.8413 (± 0.0438)	0.8658 (± 0.0256)	0.8454 (± 0.0411)
*K*NN	0.7905 (± 0.0337)	0.7465 (± 0.0578)	0.7319 (± 0.0471)	0.7378 (± 0.0514)
H*K*NN	0.8920 (± 0.0191)	0.8682 (± 0.0311)	0.8671 (± 0.0297)	0.8674 (± 0.0299)
DT	0.936 (± 0.0179)	0.937 (± 0.0250)	0.936 (± 0.0271)	0.936 (± 0.0257)
RF	0.9460 (± 0.0135)	0.9330 (± 0.0171)	0.9349 (± 0.0215)	0.9337 (± 0.0189)
AdaBoost	0.9255 (± 0.0143)	0.9120 (± 0.0128)	0.9055 (± 0.0251)	0.9082 (± 0.0194)
GB	0.9450 (± 0.0127)	0.9323 (± 0.0174)	0.9334 (± 0.0199)	0.9326 (± 0.0178)
NB	0.7374 (± 0.0300)	0.7040 (± 0.0200)	0.5882 (± 0.0214)	0.5833 (± 0.0375)
MLP	0.8938 (± 0.0188)	0.8860 (± 0.0211)	0.8478 (± 0.0436)	0.8623 (± 0.0338)
LDA	0.8715 (± 0.0139)	0.8476 (± 0.0149)	0.8375 (± 0.0098)	0.8420 (± 0.0112)
XGBoost	0.9385 (± 0.0116)	0.9289 (± 0.0130)	0.9196 (± 0.0227)	0.9237 (± 0.0173)

All classifiers performed well on our data by scoring at least 70% accuracy, and high performance as indicated by the other metrics as can be seen from Table 3. Interestingly, it is evident that DT, RF, AdaBoost, XGBoost, and GB achieved perfect scores across all metrics, showcasing their exceptional performance in identifying amebiasis cases without even the need for optimization and parameter tuning. Meanwhile, *K*NN, LDA, and NB displayed comparatively lower scores, suggesting a potential need for further optimization or inconsideration for our classification task. These results showcase the varying performance across models and provide valuable insights for selecting the most suitable classifier for the given dataset, and this justifies our comparison of a large set of classifiers to find the best one(s). In terms of theory, DT, RF, AdaBoost, XGBoost, and GB utilize decision trees as their base classifiers. Decision trees are the building blocks of these models, and they are either directly utilized (as in DT and RF) or used as base learners in boosting algorithms like AdaBoost and gradient boosting. Therefore, it is tempting to look at the decision tree resulting from the training process. Table 6 presents the decision rules that are extracted from the output decision tree of the DT classifier. As can be seen from Table 6, the resultant decision tree is used to classify cases almost accurately based mainly on strong factors such as diarrhea-RBC, diarrhea-WBC, mucus, and neutrophils (%), which are shown to be critical for diagnosing amebiasis from other non-amebiasis cases.

In summary, the decision tree alone or as part of another ensemble approach seems to effectively categorize cases into amebiasis and non-amebiasis on the basis of the aforementioned strong factors, with high accuracy. This suggests a strong predictive power of these factors in diagnosing amebiasis in the five decision tree-based models. This is also supported by the results shown in Fig. 1b, where these factors were selected as the best factors among all the other factors in our dataset, this includes the feature selection approach and the correlation of these variables to the amebiasis-dependent variable.

**Fig. 6:**
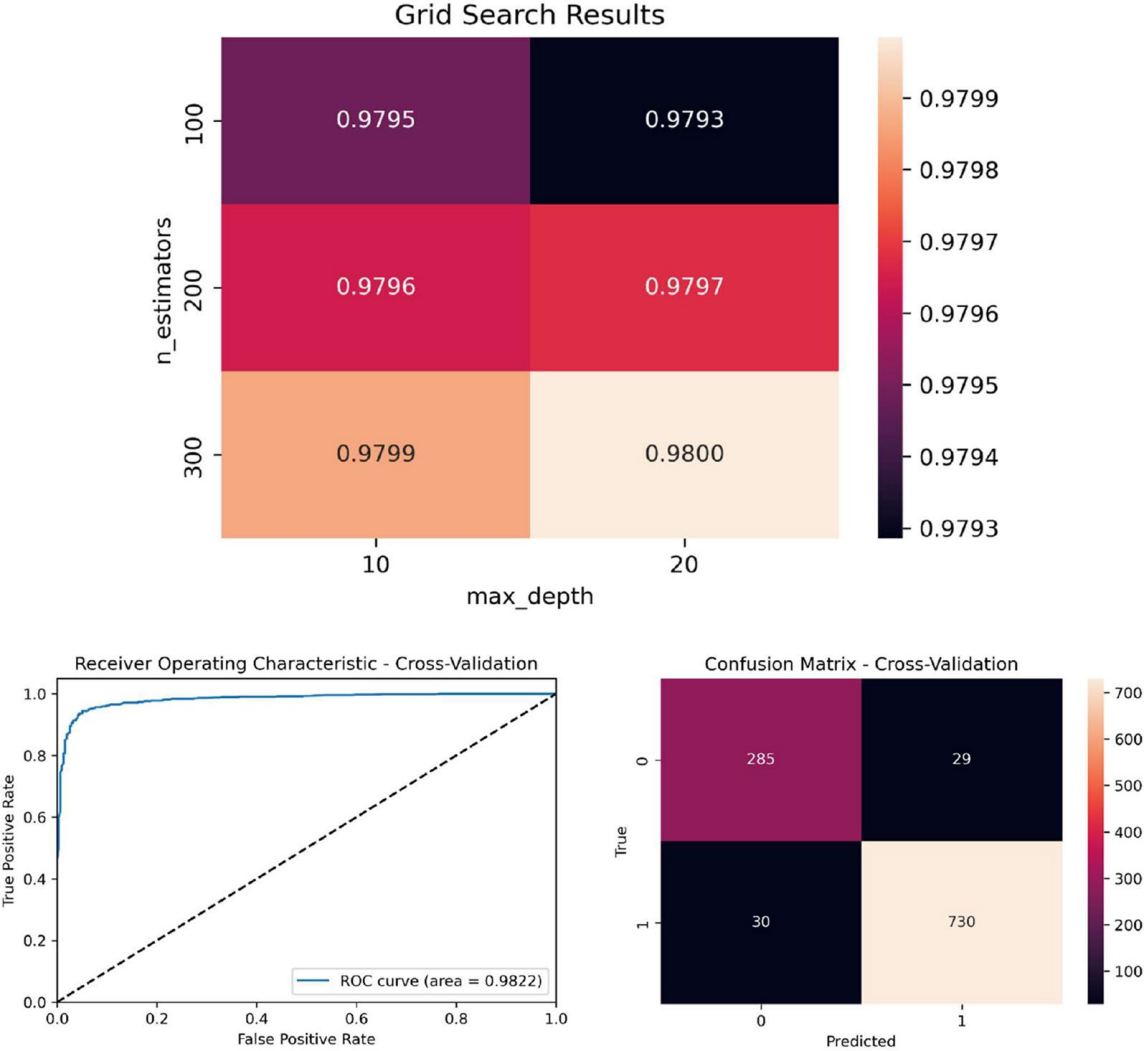
Grid search process to find the best parameters for RF (top). AUC = 0.98 results when using RF with its best parameters (bottom left), and the confusion matrix results using RF with its best parameters (bottom right)

Using its default parameters (max-depth = None, number estimators = 100), The RF classifier was the best performer in all measures, with a relatively small value of its standard deviation along the five runs of the fivefold cross-validation; therefore, it was worth fine-tuning its parameters to attempt to achieve the best performance. Figure 6 shows a grid search to find the best parameters for RF using AUC as an optimizer.

The grid search results show that the RF can perform better on our data when using 300 estimators, and the maximum depth of each tree/estimator = 20, showing a very high area under curve (AUC = 0.98) when using these parameters. The classification results using these parameters are presented in Table 4.

**Table 4:** Classification results of RF (max-depth = 20, number estimators = 300) using fivefold cross validation

Class	Accuracy	Precision	Recall	F1 score	Support
Non-amebiasis (0)	0.90	0.90	0.91	0.91	314
Amebiasis (1)	0.96	0.96	0.96	0.96	760
Weighted average	0.95	0.95	0.95	0.95	1074

Even though we used fivefold cross-validation, which guarantees that each data sample is trained and assessed over many runs, it is more robust to validate the proposed model on a fresh dataset serving generalizability. However, finding a dataset whose properties exactly matched ours was found to be quite challenging. Furthermore, considering the possible harm that Monte Carlo simulations and other oversampling approaches may do to the integrity of medical data, we are hesitant to use them to synthesize new data for extra validation (see Refs. [45, 46]).

In light of these limitations, we chose to use a holdout set to supplement the findings from our fivefold cross-validation as an alternate validation technique. This method offers a workable option in the event that an external dataset is not available, in addition to enabling us to validate the model’s performance on unseen data [53].

Table 5 illustrates how we used many training–test splits to validate our model’s robustness. In particular, we tested with several ratios, such as 10% training and 90% test, 20%, 30%, 40%, and up to 90% training with the remaining percentage serving as a test set. We were able to verify the model’s performance under various circumstances and sample sizes by iteratively adjusting the training set size.

It is expected to see higher results as the size of the training set gets larger. However, even with a very small training sample (10%), the new validation scores were consistently excellent. This result demonstrates our model’s generalizability and implies that it retains its potent predicting powers in spite of changes in the volume of training data. The efficacy of our method as a viable substitute for traditional validation is further supported by the results of our experiments, which demonstrate that even with only 10% of the data used for training (i.e., 'little training data') and 90% for testing, we achieved excellent performance. This finding highlights the generalizability of our method without the need to rely on external datasets.

**Table 5:** Results of various training–test splits to validate our model’s robustness using the optimized parameters of the RF classifier

Training %	Testing %	Accuracy	Precision	Recall	F1 score
10	90	0.908	0.914	0.908	0.910
20	80	0.925	0.925	0.925	0.925
30	70	0.936	0.938	0.936	0.937
40	60	0.927	0.929	0.927	0.928
50	50	0.933	0.934	0.933	0.933
60	40	0.942	0.942	0.942	0.942
70	30	0.957	0.957	0.957	0.957
80	20	0.977	0.979	0.977	0.977
90	10	0.981	0.981	0.981	0.981

As can be seen from Table 4, the RF benefited from the parameter tuning process, slightly increasing its accuracy by about 0.04%, and interestingly, increasing its precision, recall, and F1 score by at least 1.5%. As a result, we recommend using the RF with max-depth = 20 and number estimators =300 as being the relatively best performer among all the classifiers tested to identify amebiasis cases providing the data in hand.

**Table 6:** The decision rules extracted from the decision tree resultant model for determining amebiasis

Rule	Confidence (training)	Confidence (testing)
Diarrhea-RBC is high	$\frac{280}{280+3} \approx 0.989$	$\frac{131}{131+1} \approx 0.992$
Diarrhea-RBC is low, diarrhea-WBC is moderate, diagnosis is diarrhea, and mucus is present	$\frac{5}{5+0} = 1.0$	$\frac{4}{4+0} = 1.0$
Diarrhea-RBC is low, diarrhea-WBC is moderate, and diagnosis is abdominal pain	$\frac{9}{9+0} = 1.0$	$\frac{3}{3+0} = 1.0$
Diarrhea-RBC is low and diarrhea-WBC is very high	$\frac{28}{28+0} \approx 1.0$	$\frac{17}{17+1} \approx 0.944$
Diarrhea-RBC is low, diarrhea-WBC is low, and neutrophils (%) ≥ 0.61	$\frac{8}{8+1} \approx 0.889$	$\frac{8}{8+1} \approx 0.889$
Diarrhea-RBC is low and diarrhea-WBC is high	$\frac{20}{20+0} \approx 1.0$	$\frac{8}{8+0} \approx 1.0$
Diarrhea-RBC is moderate, diarrhea-WBC is moderate, neutrophils (%) ≥ 0.58, diagnosis is diarrhea, and WBC < 8.75	$\frac{15}{15+1} \approx 0.938$	$\frac{15}{15+3} = 0.833$
Diarrhea-RBC is moderate, diarrhea-WBC is moderate, neutrophils (%) ≥ 0.58, diagnosis is diarrhea, WBC ≥ 8.75, and Hb ≥ 12.05	$\frac{9}{9+3} = 0.75$	$\frac{2}{2+0} = 1.0$
Diarrhea-RBC is moderate, diarrhea-WBC is moderate, neutrophils (%) ≥ 0.58, and diagnosis is abdominal pain	$\frac{6}{6+0} = 1.0$	$\frac{3}{3+0} = 1.0$
Diarrhea-RBC is moderate and diarrhea-WBC is very high	$\frac{1}{1+0} \approx 1.0$	$\frac{2}{2+0} \approx 1.0$
Diarrhea-RBC is moderate, diarrhea-WBC is low, and neutrophils (%) ≥ 0.63	$\frac{9}{9+2} \approx 0.818$	$\frac{4}{4+1} \approx 0.8$
Diarrhea-RBC is moderate and diarrhea-WBC is high	$\frac{82}{82+5} = 0.942$	$\frac{33}{33+0} = 1.0$
Diarrhea-RBC is very high	$\frac{30}{30+0} = 1.0$	$\frac{15}{15+0} = 1.0$
Average confidence	0.949	0.947

Each rule is read as “If *rule* then amebiasis is positive”

We reduced the table size by including only the rules that pertain to identifying amebiasis, excluding the rules for non-amebiasis as shown in Table 6. It is worth noting that these rules are primarily composed of the top selected and correlated features, which in themselves can accurately identify amebiasis on the basis of the method used. We did not need to experiment on the selected features because certain machine learning methods incorporate feature selection as a built-in process. A decision tree is generated after evaluating the strength of each feature, starting with the strongest. Some methods utilize information gain, while others use the Gini index to determine the best feature to start the rule with.

The confidence in training is computed on the basis of how accurate a rule is during the training process, which is determined by the number of correctly classified amebiasis cases divided by the total number of cases classified by each rule. Training rules are expected to be more accurate because the method continues to work and train until it best fits the rules to the training data. On the other hand, the confidence in testing is usually lower because the resulting trained model is applied to unseen (test) data. Nevertheless, there was no notable distinction between the two, signaling the strong generalizability of the trained model and indicating that it has not been overfit by the training data. While the average of each confidence indicates the effectiveness of each of the training and testing phases. These types of measures might not fully represent the performance of a ML method owing to variations in the number of cases handled by each rule.

It is interesting to note that some rules achieved 100% confidence in both training and testing. For instance, the last rule, “If Diarrhea-RBC is very high then Amebiasis is positive,” accurately identified 30 cases during the training phase and 15 cases during the testing phase as amebiasis based solely on this rule. ML methods can easily provide such rules, which might be of great benefit to the medical field.

**Fig. 7:**
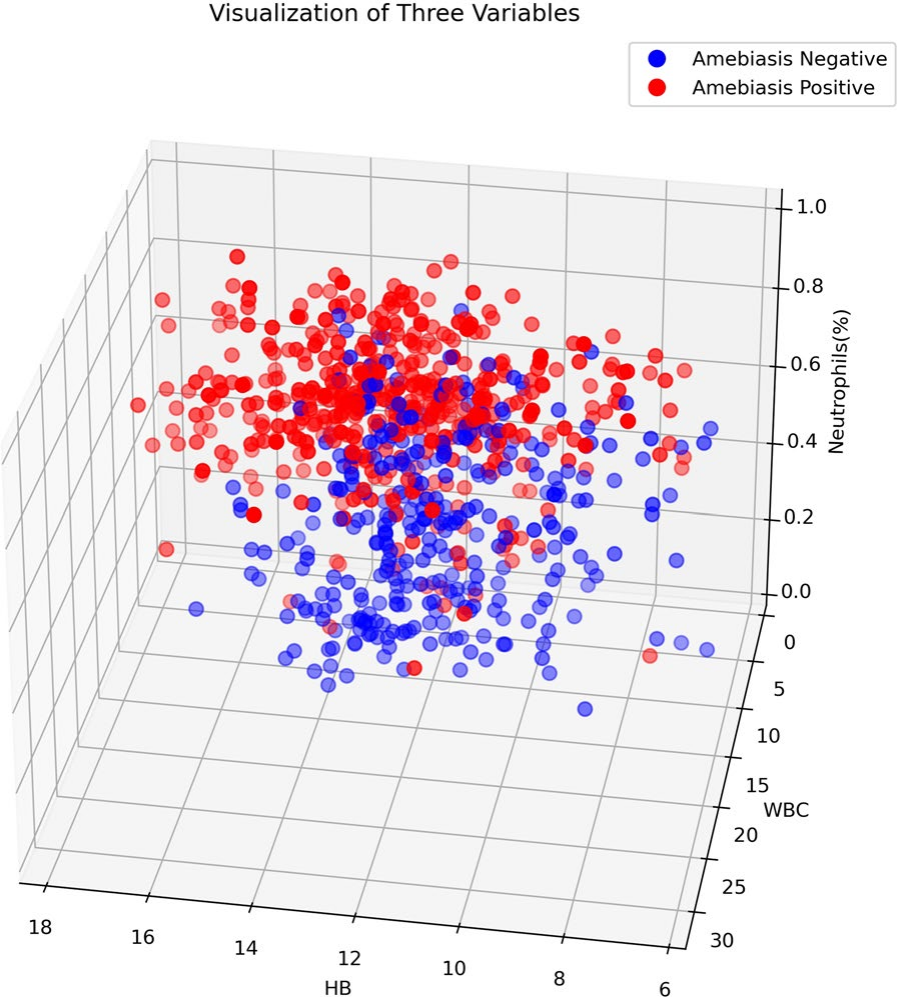
Three-dimensional visualization of three features of our data

The rules presented in Table 6 are not the sole rules that can be derived from the available data. Each decision tree produces its own set of rules, and any of these rules could be highly beneficial to the medical field. However, exploring all these rules is outside the scope of this paper.

## Discussion

On the basis of our evaluation of EMR, a significant proportion of Jordanians living in Al-Salt City are afflicted with amebiasis. It is therefore an ongoing public health issue. Conventional microscopic analysis was the main method used to detect the existence of the infection, and the diagnosis was supported by the discovery of amoebic trophozoites, cysts, or both in the specimen under examination. Verified cases of amebiasis showed signs of gastric distress, such as nausea, diarrhea, and in some cases, bloody feces. It is advised to use molecular approaches to accurately distinguish between various *Entamoeba* species, such as the pathogenic *E. histolytica* and other species, which are frequently asymptomatic and have unknown virulence, such as *E. dispar*, *E. hartmanni*, and *E. moshkovskii* [54]. However, specialized PCR-based DNA detection techniques for *E. histolytica* are only available to reference laboratories.

Previous studies conducted in various districts of Jordan demonstrated significant variability in the prevalence rate [13–15]. This figure may be related to geographical factors, such as the kind and supply of drinking water, which may affect the existence of amebiasis in different locations. According to a UNICEF report, 93% of Jordanians have access to a safe water source, and 86% have access to a piped network. In urban areas, water is typically available once a week, and less than once every 2 weeks in rural areas, with a reduced frequency during the summer. Only 77.3% of the existing sanitation systems are safely managed, and only one-third of schools have basic sanitation services [55]. This may represent an additional risk factor for cycling amebiasis in different regions of the country. Owing to the ongoing water shortage, many Jordanians drink commercially available filtered water, which could reduce the risk of contracting infection. Commercial water is typically offered in open or closed tanks in local markets in Jordan, and these tanks can be filled with reusable oxygen-sterilized containers upon request. However, how often these tanks and containers are inspected remains unclear.

In this study, we demonstrated that it is possible to predict the most crucial features of amoebic gastroenteritis using ML models based on medical records. Because it is challenging to accurately determine the exact species microscopically, the cases were identified as *Entamoeba* sp. As this is the first study about the prediction of amebiasis using ML, we focused on evaluating the suitability of algorithms using a small number of features that are chiefly derived from microscopic and hematological parameters. Corresponding to this, Sandri et al. used a few hematological markers and the naïve Bayes classifier to successfully separate toxoplasmosis patients from healthy controls [33]. We got up to 98% AUC of an improved RF model in our experiment, but the model in their instance achieved 70% AUC. We may have achieved this high performance by using a substantial quantity of data. In response to the tremendous health crisis caused by the outbreak and reemergence of infections, several research teams have created AI systems aimed at automating the identification of infectious diseases. Chadaga, for example, employed ML to differentiate between COVID-19 and non-COVID-19 pneumonia based on hematological indicators, although relatively few studies have used clinical markers as a method [41]. In comparison with our research, the investigations on malaria focused on increasing accuracy through the application of both conventional ML and DL-based algorithms [31, 56]. The use of ML for protozoal diagnostics is currently dependent on parasite detection using picture recognition [57], while our work maximizes EMR potential for ML parasitological applications. Following this inquiry, we will apply ML for *Entamoeba* species detection using microscopic images.

Finding the ideal feature combinations to include or leave out of the models is necessary. We employed the RF feature importance ranking method and cross-validation. Using this strategy allows the establishment of a more accurate and useful model to forecast amebiasis in a GE data pool. The de facto standard in conventional ML research, cross-validation, has only been used in a small number of studies [58]. The majority of the studies hold validation. In contrast, our method assesses the reliability and consistency of the features chosen across different models in use and assists in identifying features with outstanding predictive value. Prior research revealed a lack of diverse datasets, failing to include individuals with different clinical diagnoses, different laboratory results, and different demographics [57]. The development of prediction systems is severely hampered by these characteristics. The relevant studies employed a small number of ML models (e.g., Refs. [33, 34, 57]), which may induce bias and/or limit the search for more efficient ML techniques. In contrast, a variety of models were used in this study to make predictions, which produced an ideal landscape for selecting the model that best fit the available data. The predictive model would have had more statistical power if a larger sample size had been included. Nevertheless, compared with most other research that examined the suitability of ML for diagnosing infectious disorders, this analysis included a comparatively large dataset.

Leukocytosis in the stool, mucus, and red blood cells were the features relevant to the presence of amebiasis in this investigation. These findings were in excellent agreement with traditional laboratory indicators of an inflammatory illness caused by amoebic invasive infections. In actuality, the concordance between the outcomes of the ML approach and the conventional laboratory indicators serves as evidence of the ML model’s durability. Increased mucus production and stool RBCs are typically indicators of invasive enteric infections that breach the mucosa and cause tissue damage [59]. Many polymorphonuclear leukocytes (PMN) on stool microscopy suggest an inflammatory condition in the colon. Patients with amebiasis may display fecal PMNs, although they are often less numerous [60]. Generally speaking, microscopic analysis of mucus, PMN, and RBCs does not provide everything about the etiology that cannot be verified clinically. This could indicate a more serious disease such as ulcerative colitis, Crohn’s disease, or colon cancer. In comparison, using ML models combined with a feature selection technique relies on the feature’s strong correlation with class (amebiasis, non-amebiasis), while also appreciating the intercorrelation between the used features. These presumptions lead to a statistical power applied to compute each correlation. This is evident from looking at the decision rules extracted from the decision tree resultant model for determining amebiasis in (Table 6). As a result, the use of AI/ML diagnosis approaches has shown to be crucial for optimizing the outcomes of microscopic examinations that identify amebiasis infections.

In our experiments, we found that decision tree-based classifiers consistently outperformed other types of classifiers, and there are several reasons for this strong performance. First and foremost, our dataset primarily consisted of categorical variables, which we encoded using a straightforward factorization approach (e.g., assigning values such as 0, 1, 2, 3, etc.). Decision tree models, such as random forests and other types of trees, are particularly well suited for handling categorical data. They can easily split the data based on these categorical values without needing complicated transformations or assumptions about how the data are distributed, unlike models such as SVM or LDA. Our results are consistent with the work of Mathison et al. [61], who used a convolutional neural network (CNN) model to manually detect intestinal protozoa and obtained a good agreement of 98%.

Secondly, we observed considerable class overlap in our dataset, as shown in Fig. 7. This overlap posed a significant challenge for models that depend on linear decision boundaries, such as SVM and LDA, which struggled to differentiate between the classes effectively. In contrast, decision trees are nonparametric and can create nonlinear decision boundaries, allowing them to adapt better to the data’s structure, including areas where classes overlap. This flexibility enabled tree-based models to capture subtle patterns and relationships that other models missed.

Additionally, ensemble methods based on decision trees, such as random forests, gained an advantage by reducing variance through the aggregation of multiple trees, each trained on different subsets of the data. This approach not only enhanced predictive performance but also made the models more resilient to noise and variations in the dataset, improving their ability to generalize to new, unseen data.

We may be able to enhance the performance of the least-performing algorithms, such as *K*NN. Nevertheless, this paper is not intended to address such an enhancement by employing other encoding techniques, such as one-hot encoding [49], and alternative distance measurements, such as Hasanat distance, which has been shown to be unaffected by outliers and data noise [62, 63]. However, this distance was the core of H*K*NN, where the results of the *K*NN were significantly increased by at least 10% using such a distance metric; this finding is supported by Refs. [64–69], among others.

Accuracy is a statistic commonly used to assess a classifier’s overall performance in binary classification settings [45]. The F1 score is a metric that combines accuracy and recall. Still, it is also useful for evaluating a classifier’s performance in scenarios including class imbalance or where false positives and false negatives are prominent. The F1 score is especially useful when attempting to find a compromise between precision and recall, as it provides a single statistic that takes both into consideration. The F1 score thus emerges as a suitable metric for classifier comparisons in our case [70–72], taking into account the classifier’s performance on imbalanced data, even when it is slightly imbalanced as in our case, and realizing the significance in a medical application where administrators must trade off between false positives and false negatives [63]. Consequently, it was determined that the best classifier for the amebiasis prediction system was a decision tree approach.

An additional finding from this research indicates a negative correlation between WBCs and amebiasis. This finding has clinical implications because, unlike invasive bacterial infections such as *Salmonella* or *Escherichia coli* [73], leukocytoses are unexpectedly seen in amebiasis. On the other hand, neutrophil (%) correlated positively with amebiasis (0.56). In fact a sizable fraction of amebiasis cases have neutrophilic leukocytosis (Supplementary file S1: Table S1) [74]. These figures could potentially indicate the presence of a concurrent or secondary bacterial infection. However, the most common form of invasive amebiasis, amoebic liver abscess, might be associated with neutrophilic leukocytosis, along with clinical manifestations such as watery diarrhea, and less frequently with amebic colitis or even necrotizing colitis [75–77]. Hegazi et al. (2021) showed that more than 50% of hospitalized young children in Saudi Arabia had aberrant leukocytosis and neutrophilia according to age, although they did not show any amebic liver abscess on abdominal ultrasonography [20].

Clinical symptoms, such as loose stools, bleeding, and abdominal pain, along with neutrophilia, suggested that amebiasis is aggressive in this cohort and signaled the possibility of early invasive amoebic disease, requiring prompt and appropriate diagnosis and treatment. Young children and toddlers under the age of 5 years were discovered to make up more than a quarter of the patients in this study, with more than half of them having been diagnosed with watery diarrhea, which indicates a high severity of infection. This could have severe effects on children of this age as diarrheal infections linked to amebiasis are more likely to cause dehydration, stunted growth, and induce malnutrition [73, 78].

Severe complications caused by dehydration necessitate immediate hospitalization. The risk of infection is increased by inadequate breastfeeding, mixed feeding, and daycare [20]. This finding highlights the significance of closely monitoring GE in Jordanian children. It is essential to determine whether the rising prevalence of amebiasis in children is consistent in different regions across the country. Few occasional reports indicated inconsistent results. Specifically, prior surveillance research conducted in the capital city of Jordan discovered that the frequency of intestinal parasites (including *E. histolytica*) was the highest in children under the age of 5 years [14].

Additionally, children under the age of 15 years make up more than 60% of affected patients in northern Jordanian cities [15]. In contrast, adult patients in southern sites have *E. histolytica* at higher rates than children [13]. Obtaining more information on the socioeconomic situation, water quality, dietary background, nursery, and daycare is essential to understanding the causes of amebiasis in Jordan’s toddlers and young children.

This study had some limitations. The survey was performed in the Jordanian city of Al-Salt and thus focused on a single community. A more comprehensive, metacentric study involving different cohorts of patients from diverse ethnic, socioeconomic, and regional backgrounds would provide more concrete data and reproducible patterns on the distribution of potentially diverse parasite genotypes and respective differences in the pathogenesis and immune responses upon infection. However, the number of patients admitted to the city´s only hospital reflected a sufficient sample of the local population. On the one hand, information on immunological parameters—such as cytokine levels and the genotyping of the recovered *Entamoeba* spp.—would help clarify the possible causes behind the severity of amebiasis in patients from Jordan and will improve ML applications in the future.

## Conclusions

This research adds to the current application of AI technology by training existing ML-based decision tree models to identify amebiasis with greater accuracy achieved by optimization. It was possible to detect amebiasis cases with excellent accuracy. The application of ML demonstrates the technology’s ability to mine electronic medical information and trigger therapeutic action. Our methodology has the benefit of incorporating feature ranking with multiple classifiers and comparing their performance on a GE pool dataset. We used the decision tree classifier to create the best prediction system possible. A variety of laboratory tests are included in this system, with a focus on diarrhea-RBC, diarrhea-WBC, neutrophil (%), and WBC measures as the strongest features. Using the data on hand, we have depicted the epidemiological picture of amebiasis among patients with amebiasis in all age and sex categories. Large-scale research on Jordan’s amebiasis is required, as is the development of preventative strategies to lower the disease’s prevalence in high-risk settings including daycare centers and educational institutions.

## Supplementary Information

Supplementary Material. Table S1. Aberrant neutrophil (%) among the different age groups of amebiasis Jordanian cases

## Data Availability

A novel dataset supporting this study’s finding will be shared alongside the publication, and a suitable link to the data will be provided.

## Code availability

The code can be made available upon request from the last author.
