# Supplementary material for: Decision tree-based learning and laboratory data mining: an efficient approach to amebiasis testing
Source: Parasit Vectors. 2025 Jan 29;18:33. doi: 10.1186/s13071-024-06618-6 (PMC11780931; doi:10.1186/s13071-024-06618-6)
Supplement: Supplementary file 1 — Supplementary Material. Table S1. Aberrant neutrophil (%) among the different age groups of amebiasis Jordanian cases [file 13071_2024_6618_MOESM1_ESM.docx]

**Supplementary file S1: Table S1.** Aberrant Neutrophil (%) among the different age groups of amebiasis Jordanian cases

| Age group (year) | % Aberrant neutrophil* |
| --- | --- |
| 1mo-4year | 23.5 |
| 5-18 | 17.8 |
| 19-59 | 42.9 |
| ≥ 60 | 15.7 |

* abnormal neutrophil count (≥7.000 per mm3).

The Greek alphabet
